# Supplementary material for: Dose Optimization of Anxiolytic Compounds Group in Valeriana jatamansi Jones and Mechanism Exploration by Integrating Network Pharmacology and Metabolomics Analysis
Source: Brain Sci. 2022 Apr 30;12(5):589. doi: 10.3390/brainsci12050589 (PMC9138999; doi:10.3390/brainsci12050589)
Supplement: Supplementary file 1 [file brainsci-12-00589-s001.zip › brainsci-1678933-supplementary.pdf]

Supplementary Material

1. Supplementary Tables

Table S1. Uniform designed table U*8 (8^5)

	1	2	3	4	5
1	1	2	4	7	8
2	2	4	8	5	7
3	3	6	3	3	6
4	4	8	7	1	5
5	5	1	2	8	4
6	6	3	6	6	3
7	7	5	1	4	2
8	8	7	5	2	1

Table S2. Using table of U*8 (8^5)

s	Column number	D
2	1 3	0.1445
3	1 3 4	0.2000
4	1 2 3 5	0.2709

Table S3. Anxiolytic effect of ZZX-UDGs on EBS rat in behavioral test

Groupds	OE%	OT%	CE	CT
Contorl	49.06 ± 3.04	30.23 ± 2.14	11.60 ± 2.02	33.47 ± 6.13
EBS	37.50 ± 5.48$^{\#\#}$	12.57 ± 2.22$^{\#\#}$	1.70 ± 0.65$^{\#\#}$	6.80 ± 2.85$^{\#\#}$
DZP	56.23 ± 3.77**	34.03 ± 9.04**	5.50 ± 0.93*	27.59 ± 5.86*
UDG-1	48.16 ± 3.44**	26.78 ± 3.71*	5.40 ± 1.24*	25.79 ± 7.13*
UDG-2	51.25 ± 2.49**	36.19 ± 5.73**	4.40 ± 1.31	14.33 ± 4.51
UDG-3	45.51 ± 4.17*	24.70 ± 4.58	3.90 ± 0.77	15.16 ± 3.59
UDG-4	53.09 ± 4.40**	29.20 ± 6.59*	7.40 ± 1.10**	26.74 ± 3.62*
UDG-5	48.18 ± 3.35**	31.99 ± 3.88**	3.60 ± 0.70	17.40 ± 4.53
UDG-6	47.63 ± 2.25**	34.76 ± 4.70**	4.10 ± 0.84	12.65 ± 3.20
UDG-7	53.41 ± 3.08**	26.13 ± 2.40	5.20 ± 1.54*	27.46 ± 9.92*
UDG-8	53.25 ± 4.84**	27.96 ± 6.39	7.13 ± 1.14**	40.87 ± 11.56**

$^{\#}p < 0.05$, $^{\#\#}p < 0.01$ vs control group, $^{*}p < 0.05$, $^{**}p < 0.01$ vs EBS group ($n = 8 \sim 10$; mean ± SEM)

Table S4. Comparation of the anxiolytic effect in the two optimal solutions (OSs) and ethanol extract of ZZX

Groupds	OE%	OT%	Number of central entries	Time spent in central areas
Contorl	55.20 ± 4.47	27.15 ± 11.69	12.90 ± 1.39	29.51 ± 3.22
EBS	37.92 ± 5.06$^{##}$	3.77 ± 2.02$^{##}$	3.60 ± 0.87$^{##}$	7.61 ± 2.03$^{##}$
DZP	49.66 ± 2.19*	20.17 ± 4.35*	12.50 ± 2.14**	26.19 ± 4.61**
ZZX	50.72 ± 8.79	21.72 ± 12.15*	10.90 ± 1.64**	22.89 ± 4.80**
ZZX-OS1	47.50 ± 3.46*	13.98 ± 1.88	12.30 ± 1.77**	27.55 ± 5.06**
ZZX-OS2	48.77 ± 3.13*	18.91 ± 2.80*	13.10 ± 1.86**	30.03 ± 5.82**

$^{#}p < 0.05$, $^{##}p < 0.01$ vs control group, $^{*}p < 0.05$, $^{**}p < 0.01$ vs EBS group (mean ± SEM)

EPM: $n = 6 \sim 8$; OFT: $n = 10$

Table S5. Name and classification of metabolites in rat hippocampus

No.	Metabolites	Category
1	Dehydroascorbic acid	Alcohols
2	Diglycerol	Alcohols
3	Erythritol	Alcohols
4	Myoinositol	Alcohols
5	Ethanolamine	Alkylamines
6	Maleimide	Alkylamines
7	N-Acetylputrescine	Alkylamines
8	3-Aminoisobutanoic acid	Amino Acids
9	L-Alanine	Amino Acids
10	L-Allothreonine	Amino Acids
11	Beta-Alanine	Amino Acids
12	L-Glutamic acid	Amino Acids
13	Glycine	Amino Acids
14	L-Leucine	Amino Acids
15	N-Acetyl-L-aspartic acid	Amino Acids
16	N-Methylalanine	Amino Acids
17	Norvaline	Amino Acids
18	Pyroglutamic acid	Amino Acids
19	L-Phenylalanine	Amino Acids
20	L-Proline	Amino Acids
21	L-Serine	Amino Acids
22	L-Tyrosine	Amino Acids
23	Urea	Amino Acids
24	Lithocholic acid	Bile Acids
25	1-Deoxyerythritol	Carbohydrates
26	3-Phosphoglyceric acid	Carbohydrates

27	D-Arabitol	Carbohydrates
28	Cellobiose	Carbohydrates
29	Fructose-6-phosphate	Carbohydrates
30	D-Galactose	Carbohydrates
31	Galactose-6-phosphate	Carbohydrates
32	Gluconolactone	Carbohydrates
33	D-Glucose	Carbohydrates
34	Mannose-6-phosphate	Carbohydrates
35	N-Acetylmannosamine	Carbohydrates
36	Sorbitol	Carbohydrates
37	Sucrose	Carbohydrates
38	Erythrose	Carbohydrates
39	Arachidonic acid	Fatty Acids
40	Behenic acid	Fatty Acids
41	11Z-Eicosenoic acid	Fatty Acids
42	DHA	Fatty Acids
43	Dodecanoic acid	Fatty Acids
44	Oleic acid	Fatty Acids
45	Palmitic acid	Fatty Acids
46	Palmitoleic acid	Fatty Acids
47	Pentadecanoic acid	Fatty Acids
48	Stearic acid	Fatty Acids
49	Sulfate	Inorganic Oxide
50	1-Monoolein	Lipids
51	2-Monostearin	Lipids
52	Glycerol-3-phosphate	Lipids
53	Zymostrol	Lipids
54	Adenine	Nucleotides
55	Adenosine	Nucleotides
56	AMP	Nucleotides
57	Hypoxanthine	Nucleotides
58	UDP-N-acetylglucosamine	Nucleotides
59	Uracil	Nucleotides
60	Uridine	Nucleotides
61	3-Hydroxybutyric acid	Organic Acids
62	Ureidopropionic acid	Organic Acids
63	Benzoic acid	Organic Acids
64	Glyceric acid	Organic Acids
65	L-Lactic acid	Organic Acids
66	Malonic acid	Organic Acids
67	Methylphosphate	Organic Acids

68	Pyruvic acid	Organic Acids
69	Succinic acid	Organic Acids
70	Ascorbic acid	Vitamins
71	Niacinamide	Vitamins
72	Alpha-Tocopherol	Vitamins
73	1-Monostearin	Unknown

Table S6. Effect of ZZX-ACG on action targets of differential metabolites in hippocampus of anxiety rats

No.	Description	Gene symbol
1	acyl-CoA thioesterase 8	ACOT8
2	acyl-CoA thioesterase 2	ACOT2
3	solute carrier family 27 (fatty acid transporter), member 2	SLC27A2
4	cytochrome P450, family 4, subfamily F, polypeptide 8	CYP4F8
5	acyl-CoA thioesterase 7	ACOT7
6	acyl-CoA thioesterase 4	ACOT4
7	phospholipase A2, group IVE	PLA2G4E
8	cytochrome b5 domain containing 1	CYB5D1
9	cytochrome P450, family 1, subfamily A, polypeptide 1	CYP1A1
10	cytochrome P450, family 1, subfamily A, polypeptide 2	CYP1A2
11	cytochrome P450, family 1, subfamily B, polypeptide 1	CYP1B1
12	cytochrome P450, family 2, subfamily A, polypeptide 6	CYP2A6
13	cytochrome P450, family 2, subfamily A, polypeptide 7	CYP2A7
14	cytochrome P450, family 3, subfamily A, polypeptide 7	CYP3A7
15	cytochrome P450, family 2, subfamily A, polypeptide 13	CYP2A13
16	cytochrome P450, family 2, subfamily B, polypeptide 6	CYP2B6
17	cytochrome P450, family 2, subfamily C, polypeptide 8	CYP2C8
18	cytochrome P450, family 2, subfamily C, polypeptide 9	CYP2C9
19	cytochrome P450, family 2, subfamily C, polypeptide 18	CYP2C18
20	cytochrome P450, family 2, subfamily D, polypeptide 6	CYP2D6
21	cytochrome P450, family 2, subfamily E, polypeptide 1	CYP2E1
22	cytochrome P450, family 2, subfamily F, polypeptide 1	CYP2F1
23	cytochrome P450, family 2, subfamily J, polypeptide 2	CYP2J2
24	cytochrome P450, family 3, subfamily A, polypeptide 4	CYP3A4
25	cytochrome P450, family 3, subfamily A, polypeptide 5	CYP3A5
26	cytochrome P450, family 4, subfamily A, polypeptide 11	CYP4A11
27	cytochrome P450, family 4, subfamily B, polypeptide 1	CYP4B1

28	cytochrome P450, family 19, subfamily A, polypeptide 1	CYP19A1
29	lactate dehydrogenase A-like 6A	LDHAL6A
30	cytochrome P450, family 4, subfamily Z, polypeptide 1	CYP4Z1
31	acyl-CoA synthetase long-chain family member 1	ACSL1
32	acyl-CoA synthetase long-chain family member 3	ACSL3
33	acyl-CoA synthetase long-chain family member 4	ACSL4
34	MYC binding protein 2	MYCBP2
35	acyl-CoA synthetase long-chain family member 6	ACSL6
36	arachidonate 12-lipoxygenase	ALOX12
37	arachidonate 5-lipoxygenase	ALOX5
38	arachidonate 15-lipoxygenase	ALOX15
39	arachidonate 15-lipoxygenase, type B	ALOX15B
40	phospholipase A2, group IVF	PLA2G4F
41	cytochrome P450, family 4, subfamily X, polypeptide 1	CYP4X1
42	phospholipase A2, group IID	PLA2G2D
43	phospholipase A2, group IVD (cytosolic)	PLA2G4D
44	cytochrome P450, family 4, subfamily A, polypeptide 22	CYP4A22
45	cytochrome P450, family 2, subfamily S, polypeptide 1	CYP2S1
46	phospholipase A2, group IIE	PLA2G2E
47	lactate dehydrogenase A	LDHA
48	lactate dehydrogenase B	LDHB
49	lactate dehydrogenase C	LDHC
50	phospholipase A2, group III	PLA2G3
51	peptidylglycine alpha-amidating monooxygenase	PAM
52	acyl-CoA synthetase long-chain family member 5	ACSL5
53	6-phosphofructo-2-kinase/fructose-2,6-biphosphatase 1	PFKFB1
54	6-phosphofructo-2-kinase/fructose-2,6-biphosphatase 2	PFKFB2
55	phospholipase A2, group IB (pancreas)	PLA2G1B
56	phospholipase A2, group IIA (platelets, synovial fluid)	PLA2G2A
57	phospholipase A2, group IVA (cytosolic, calcium-dependent)	PLA2G4A
58	phospholipase A2, group V	PLA2G5
59	bile acid CoA: amino acid N-acyltransferase	BAAT
60	prostaglandin-endoperoxide synthase 1	PTGS1
61	prostaglandin-endoperoxide synthase 2	PTGS2
62	cytochrome P450, family 4, subfamily F, polypeptide 11	CYP4F11
63	acyl-CoA thioesterase 1	ACOT1

64	acyl-CoA thioesterase 6	ACOT6
65	phospholipase A2, group IIF	PLA2G2F
66	cytochrome P450, family 3, subfamily A, polypeptide 43	CYP3A43
67	cytochrome P450, family 4, subfamily F, polypeptide 12	CYP4F12
68	phospholipase A2, group XIIA	PLA2G12A
69	phospholipase A2, group VI (cytosolic, calcium-independent)	PLA2G6
70	phospholipase A2, group X	PLA2G10
71	phospholipase A2, group XIIB	PLA2G12B
72	phospholipase A2, group IVC (cytosolic, calcium-independent)	PLA2G4C
73	lactate dehydrogenase A-like 6B	LDHAL6B

Table S7. VIP result data of metabolites in OPLS-DA

No.	Metabolites	VIP	Corr.Coeffs.
1	Cellobiose	2.108284	0.864111
2	Dehydroascorbic acid	2.101373	0.861279
3	Pentadecanoic acid	2.038412	−0.83547
4	L-Lactic acid	1.91941	−0.7867
5	Gluconolactone	1.770639	−0.72572
6	D-Arabitol	1.68665	0.691299
7	Behenic acid	1.675166	−0.68659
8	AMP	1.667638	0.683506
9	N-Methylalanine	1.589427	−0.65145
10	Arachidonic acid	1.580077	0.647618
11	Methylphosphate	1.514223	0.620626
12	DHA	1.46647	−0.60105
13	Palmitoleic acid	1.414276	0.579662
14	Sulfate	1.404503	−0.57566
15	3-Hydroxybutyric acid	1.376367	−0.56412
16	Pyruvic acid	1.342514	0.550249
17	L-Glutamic acid	1.333052	−0.54637
18	Fructose-6-phosphate	1.291421	0.529308
19	Erythrose	1.197031	0.490621
20	Adenine	1.18926	−0.48744
21	1-Monoolein	1.184344	−0.48542
22	Glycerol-3-phosphate	1.094798	0.448719
23	Ethanolamine	1.073188	0.439862
24	L-Alanine	1.026825	0.420859
25	Pyroglutamic acid	1.012105	0.414826

26	D-Galactose	1.006881	−0.41269

2. Supplementary Figures

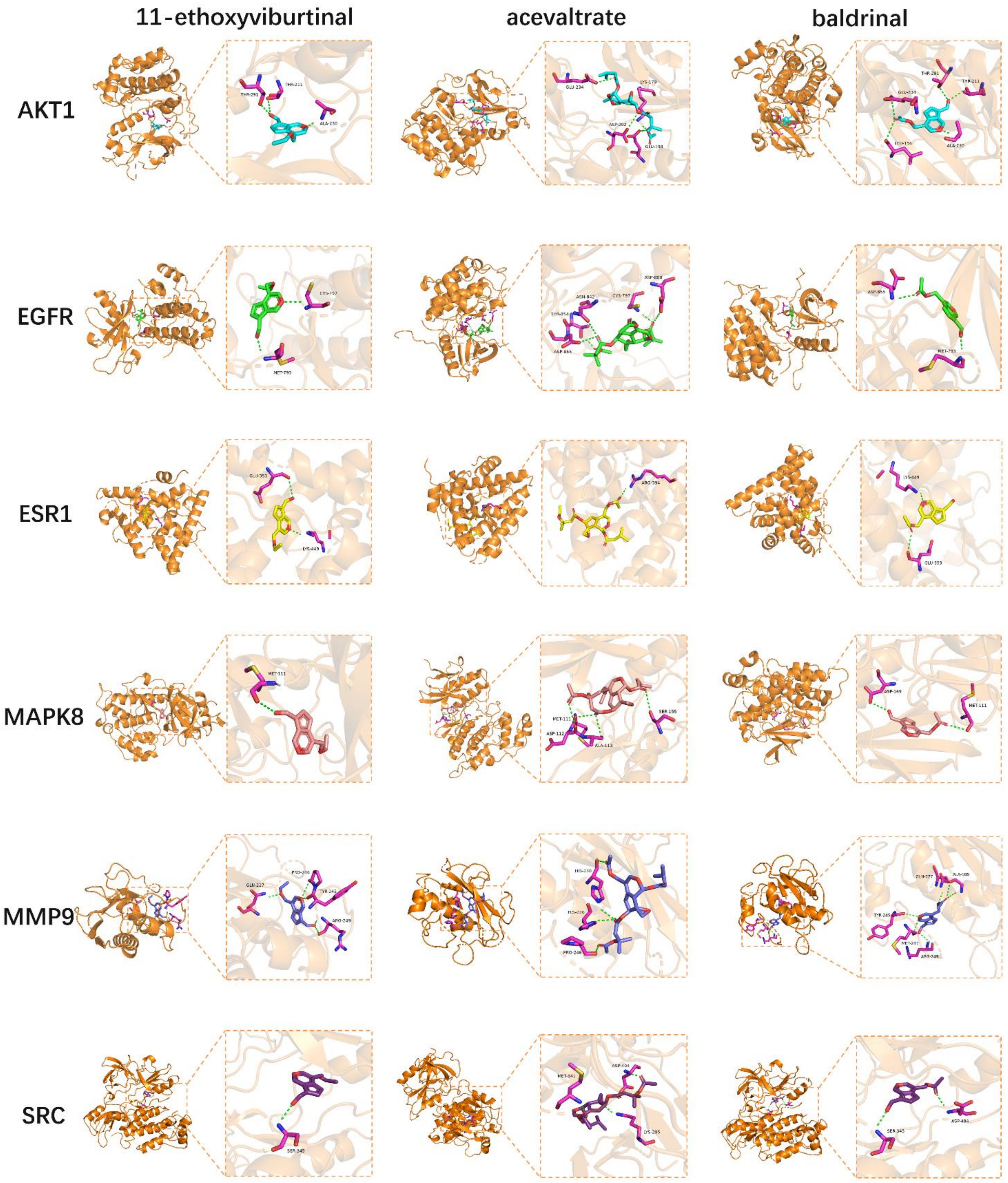

Figure S1. Molecular docking analysis of 3 compounds and 6 core target proteins.
